# Does the board’s on-site decision inhibit over-investment

**DOI:** 10.1371/journal.pone.0255453

**Published:** 2021-08-05

**Authors:** Xiaofei Shi, Fei Zhao, Long Xu, Na Bian, Fengfei Wang

**Affiliations:** 1 School of Business Administration, Hebei University of Economics and Business, Shijiazhuang, Hebei, China; 2 School of Management, Hebei University of Geosciences, Shijiazhuang, Hebei, China; Taiyuan University of Science and Technology, CHINA

## Abstract

It is an effective expansion of the research on the Board of Directors to do the research based on different board meeting forms and their effects sampling A-share companies listed in 2007–2017, the article empirically tests the impact of the times of board meetings, the proportion of on-site board meetings on listed companies’ over-investment. Consequently and significantly, the times of board meetings is positively correlated with over-investment, while the proportion of on-site board meetings is negatively correlated with over-investment. That is, the on-site meeting for the Board decision-making will better inhibit the enterprises’ over-investment behaviors. Further research shows that when there is a controlling shareholder in the company or in a dual position, the on-site board meeting no longer has a significant inhibitory effect on over-investment. By research on the independence of the Board of Directors, it is found that when selecting on-site board decision-making, the existence of independent directors has an over-investment suppression effect, and the higher the proportion of independent directors, the more obvious the inhibitory effect is. The samples are divided into state-owned enterprises and private enterprises, the study found that when choosing on-site board meetings, state-owned enterprises have a greater inhibitory effect on over-investment than private enterprises. The findings of this study will enrich the research of the board meeting and provide a new testing method for the relevant research of the Board of Directors.

## Introduction

**Fig 1:**
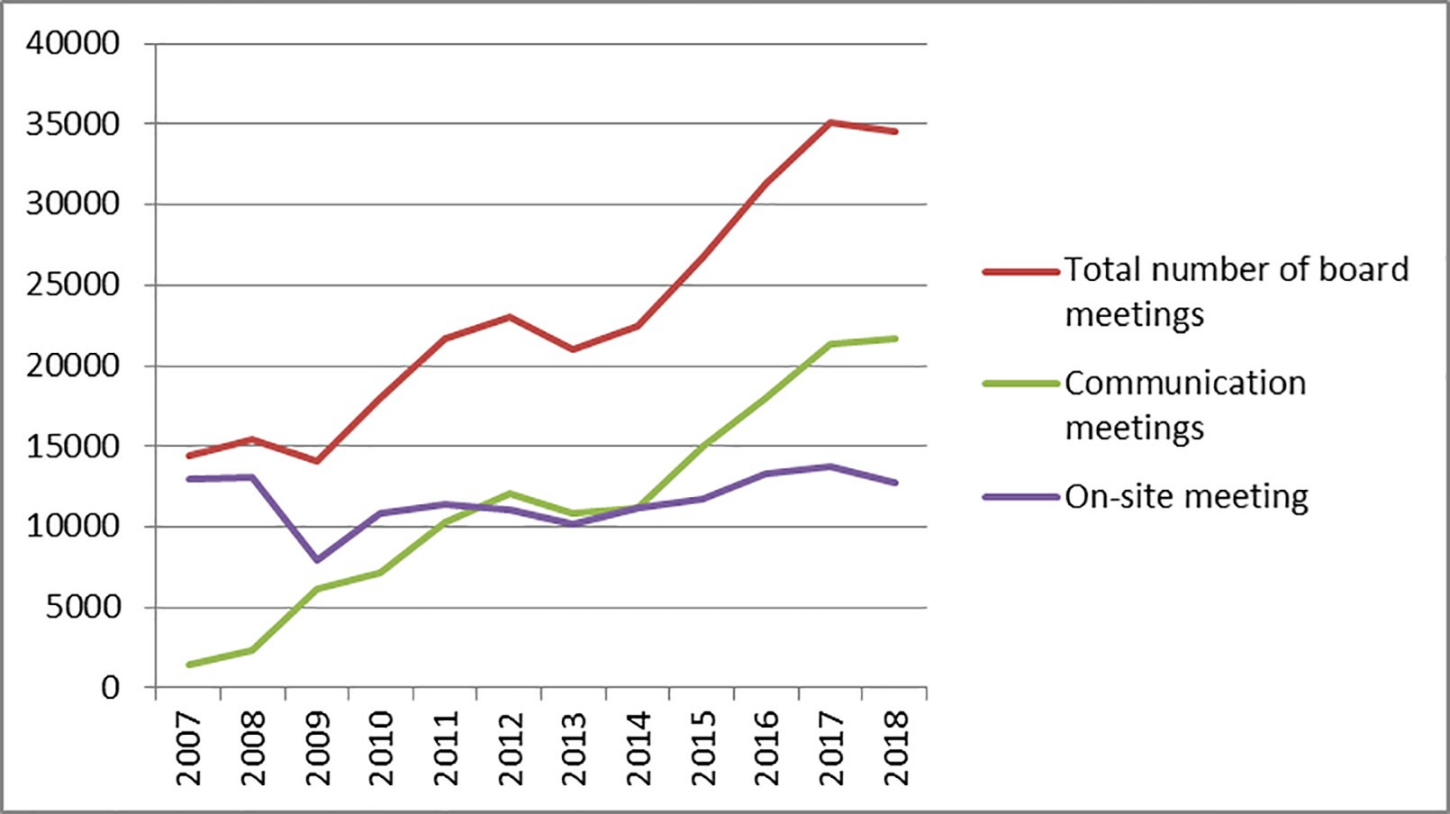
Number of the board meetings.

**Fig 2:**
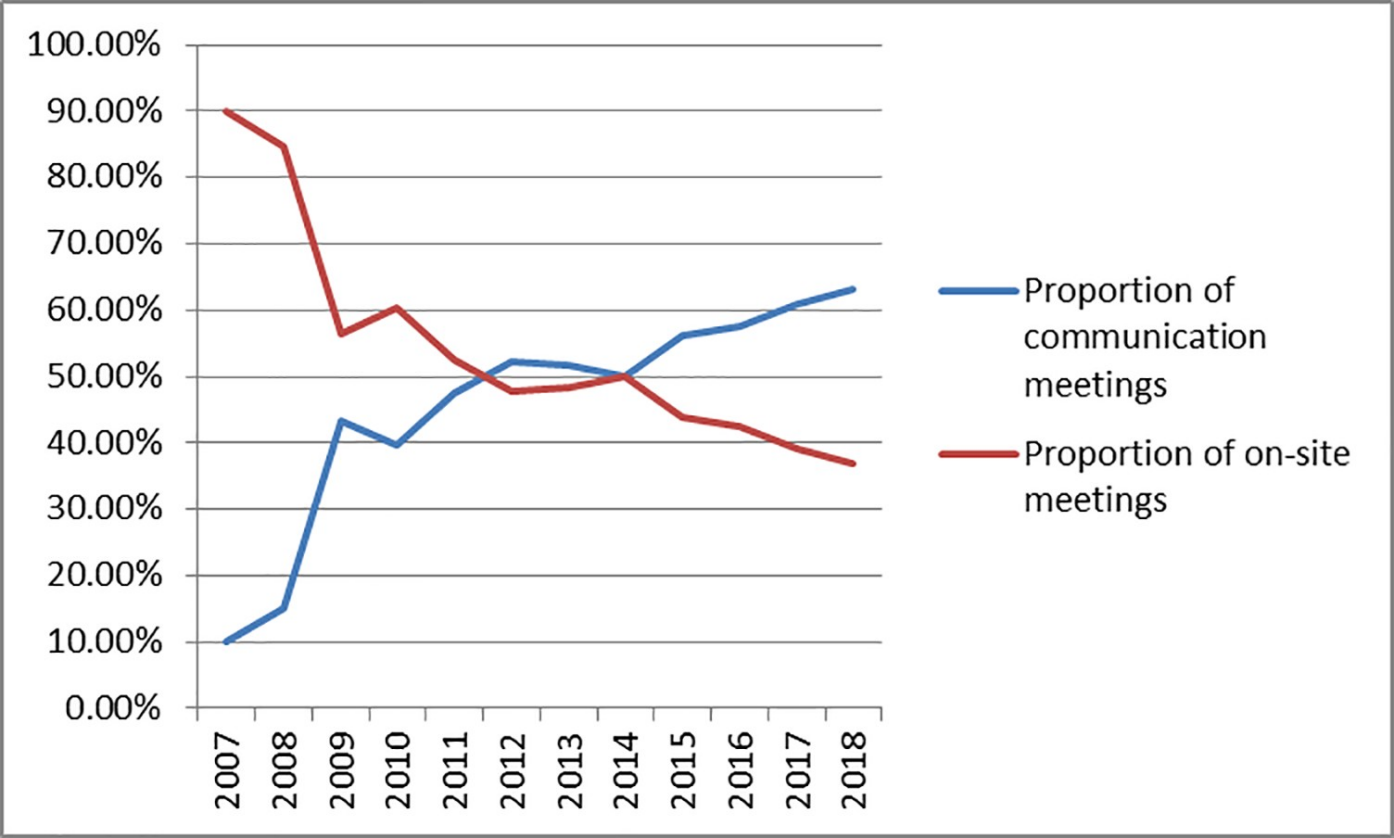
Proportion of the board meetings.

With the development of internet technology and its applications, the communication meetings (The communication meeting refers to the meeting of the board of directors held by means of communication, such as video, telephone, or letter) are widely used in the board meeting of the listed companies. When choosing the meeting form of the Board of Directors in the company law of China, there is no clear requirement for the meeting form. As a more convenient form of decision-making at the board meetings, the communication voting mode of the Board of Directors is more and more favored by the company. According to the statistics of the CCER database and the data of relevant websites such as Sina.com., Dongfang Eastmoney.com., and China Financial Information. Many listed companies are increasingly inclined to choose the form of the communication meetings when a board meeting is held in 2007–2018 (The Figs 1 and 2). In 2011, the proportion of the communication meetings exceeded that of on-site meetings (The on-site meeting refers to the meeting of the board of directors held in the form of on-site face-to-face).

The Board of Directors is the core of corporate governance which is a coordinator of conflicts of interest between the company owners and managers, and plays a decisive role in the company’s business strategy [1]. It makes decisions and supervises managers. Board decision-making is a special form of group decision-making [2, 3]. Board meetings are the main manifestation of the board governance mechanism and the important tool for the board to play the role in the corporate governance [4]. In a sense, the number of board meetings reflects the strength of the board’s behavior from one side [5]. Through the current research on board meetings at home or abroad, it is found that most of the literature focuses on the impact of the number of board meetings on corporate performance. The main points are as follows: one view is that the aim of board meetings is conducive to improving the company’s performance. Directors who meet frequently and have sufficient communication may know more about the company’s information and make more efficient decisions. Therefore, the higher the number of board meetings, the communication between the directors will be more sufficient, which will be helpful to improve the company’s performance [6, 7]. Another view is that board meetings are useless, because they are not used to exchange useful information with each other between managers in the limited time when all the directors come together. It is found that the number of board meetings has no significant impact on the company’s performance [8, 9]. Other scholars believe that the high number of board meetings is likely to be a response of the company to poor operating performance. Board meetings are like “the fire extinguishers” rather than the preventive devices [8, 10].

The Board of Directors is the very important internal oversight and control mechanism in the corporate governance [8]. Board meetings are the main method of Board of Directors to work, and it is an important way for directors to participate in the decisions and understand the situation of the company [11]. The effectiveness of board meetings plays a vital role in the company’s development [12, 13]. This may be due to the low cost of the communication meetings, which can ensure that all directors can attend the meeting in person and participate in voting. However, the excessive use of the communication meetings in board meetings may also be detrimental to the communication between directors and reduce the effectiveness of board decisions. The impact of different board meetings on the company requires more in-depth research. The investment is one of the most important economic activities of the company [9], and it is also a common indicator for testing the effectiveness of the corporate governance. This research uses the indicator of over-investment of the companies to test the impact of the board meetings format on the company’s investment decision. Based on the research logic, this research first conducts an empirical test of the relationship between the number of board meetings and the company’s over-investment. Then it focuses on examining the relationship between the proportion of on-site meetings and the company’s over-investment. Considering the differences of important corporate governance variables, it further examined the impact of the proportion of on-site meetings on the company’s over-investment in the case of differences in controlling shareholders, the CEO duality, the independence of the board, and the nature of equity.

The contributions of this research: (1) Most of the previous literatures have focused on the board structure and directors’ characteristics, such as the characteristics of independent directors [11], Female director [14], the CEO duality [15]. Our research will follow the logic from "structure" to "behavior" and further expand to the impact of board meeting decision-making on the over-investment. It will expand the relevant research ideas of the Board of Directors. (2) Most domestic and foreign literatures have examined board meetings as a control variable or a certain aspect of board characteristics [8, 16–18], or directly test the impact of the board meeting number on company performance [5, 8]. This research will focus on expanding the board meeting number to the board meetings format. It will further expand the research of the board meeting decision-making. (3) Board meetings are very important behavior that affect the company’s investment [2, 18–20]. Our research discusses the logical relationship between the form of board meetings and the decision of Board of Directors. It focuses on the impact of the on-site meetings form of the Board of Directors on the over-investment decision-making of the company, and will be a new test path for the research from the board actions to the company performance.

## Literature reviews and research hypotheses

### The board meetings and the over-investment

The Board of Directors exercises its functions and powers through the board meetings. The board meetings are a very specific and key issue in the modern corporate management and corporate governance. It is an important way to ensure directors’ participation in decision-making and understand the strategies of company [11]. It’s also an important sign to measure the intensity of directors’ performance of duties [21] and the key to determine the company performance [5]. The Principal-Agent Theory holds that the Board of Directors bears the dual responsibilities of trust and commission in the company. On the one hand it is entrusted by the shareholders’ meeting and assumes the fiduciary responsibility, on the other hand it is entrusted to the managers. In the arrangement of the governance structure of listed companies, the Board of Directors is an important link between shareholders and managers. It influences and decides the major decisions of the company’s production and operation to a certain extent. For example, according to the law of company in China, the Board of Directors has the right to call the general meeting of shareholders, implement the resolutions of the general meeting of shareholders, make major decisions on the investment, financing, and employ or dismiss the managers. In the actual production and operation, the managers are responsible to the Board of Directors. In the Board of Directors, all decision-making is usually made through board meetings and all directors voting. The higher the board meetings number, the more things the company needs to make decisions. The investment decision is an important decision of the company. Therefore, according to the logic, the number of board meetings directly affects the company’s investment decisions.

The separation of ownership and control makes a large number of agency problems unavoidable in modern companies. Managers may not make decisions in the interests of shareholders. According to Principal-Agent Theory and Information Asymmetry Theory, managers are more likely to use information advantage. Jensen and Meckling [16] have found that the managers’ hard work results are shared by shareholders, while the cost is borne solely by managers. It inevitably leads to that the managers’ decision-making is not to maximize the shareholders’ interests, but it is more beneficial to their own interests. The most direct way for managers to obtain private benefits is to expand the size of the company [9]. Therefore, the managers are more willing to invest. Jensen [22] pointed out that the schedule of board meetings is mostly determined by the CEO. The routine procedure occupied the time for the outside directors to exercise their control over the managers, which reduced the usefulness of the communication between the directors and between directors and managers. It limited the effective supervision of the outside directors over the managers. Jensen [22] and Vafeas [8] point out that when the number of board meetings exceeds the necessary, the board meetings will be inefficient and detrimental to the performance of the company. It can be seen that when the number of board meetings exceeds a certain number, the efficiency of board meetings may have been affected, and the more likely the company is to overinvest.

Based on the above analysis, we formulate the following:

Hypothesis 1a: There is a positive relationship between the number of board meetings and the over-investment.
Hypothesis 1b: There is a positive relationship between the number of communication meetings and the over-investment.
Hypothesis 1c: There is a positive relationship between the number of on-site meetings and the over-investment.

### The proportion of on-site meetings and the over-investment

The Board of Directors is the core of corporate governance. The purpose of corporate governance is not only to build the effective governance structure, but also to implement scientific decisions through the effective governance mechanisms. Board decision making is a special form of the group decision making [23]. The performance of a company depends heavily on the quality of its decisions, so the importance of board decisions should be given full attention [23]. As a decision-making team, cognitive conflicts among board members are inevitable, and the sufficient cognitive conflicts help promote the information exchange between members [17]. It promotes directors to consider and evaluate alternatives [18], improves the quality of team discussions [20]. It forces teams to carefully review task-related information [24]. We can use the full board meetings to reduce the friction between directors and managers and enhance mutual trust. The members can reduce opportunistic behavior through the trust and obedience [25].

Based on the theory of information asymmetry, for a group decision-making organization, an important feature of making effective decisions is the ability to integrate the relevant information mastered by decision-making members and apply it to the final decision-making. That is, when some important information is mastered by different members, an effective group decision-making organization needs communication and information sharing among members, So that information can be effectively mastered by all members [26, 27]. Board meetings are the most important channel for directors to gather information, implement decisions, and supervise managers [28]. Lipton and Lorsch [6] pointed out that it’s a problem for directors is the lack of time to perform tasks in practical work. If the opportunity to meet between directors is increased, the time for communication and exchange between directors will be increased accordingly. The communication will improve the quality of discussions, and obviously improve the effectiveness of the governance of board [7]. For directors, meeting often can better exercise their rights. The face-to-face communication will be more effective in information acquisition and decision-making accuracy [29]. Because the face-to-face communication can not only obtain a lot of information, but also reap the benefits of nonverbal communication [30]. The information expressed by many body language is also very important, while some of them subconsciously, and none of them are meaningless [29]. The face-to-face communication is more conducive to information sharing and can also get instant feedback. Some important information should be held by different board members. The efficient board meetings require the communication and information sharing between the directors, so that the information can be effectively controlled by all directors. The way of the on-site meetings are more conducive to the sharing of information among directors than the communication meetings. And the face-to-face communication, people will spend more time on the analysis and reasoning, so the directors can have a clearer understanding of the company’s operating conditions and make better investment decisions.

Modern organizational theory holds that the Board of Directors is an organization with strategic decision-making as its core. To participate in strategic decision-making, supervise managers, and control the CEO is the key function of the Board of Directors [31]. The on-site meetings are also a manifestation of the board’s strong supervision. Directors who meet frequently may better fulfill their responsibilities, so that the managers can act in accordance with and maximize shareholders’ interests. The supervision of directors weakened the agency behavior of the managers and the degree of information asymmetry, thereby reduced the opportunistic behavior of the managers. In this way, the managers can reduce the blind investment behavior, so as to alleviate the excessive investment of the listed companies. It can be reasonably inferred that the greater the proportion on-site meetings of the Board of Directors, the more willing the directors to perform their duties which related to the interests of the shareholders.

Based on the above analysis, we formulate the following:

Hypothesis 2: There is a negatively relationship between the proportion of the on-site meetings and the over-investment.

## Research design

### Sample selection

Our samples are initially composed of all listed companies on the Shanghai Stock Exchange (SHSE) and Shenzhen Stock Exchange (SZSE) from 2007 to 2018. The data of this study were derived from the CSMAR and CCER database. The relevant data of the board meetings come from CSMAR’s the governance structure research database of China listed company and CCER database; the relevant data for calculating over-investment are from the CSMAR database. In order to ensure the quality of the data, the effective samples are determined based on the general principles of domestic research. The following samples are excluded: (1)The financial and insurance listed companies; (2)The companies whose data cannot be obtained during the sample study; (3)The number of board meetings data below two times; (4)The companies’ asset-liability ratio more than 1; (5)The companies with special transaction status such as ST or * ST; (6)The samples missing financial data. Finally, we obtain valid 23159 samples. In order to overcome the possible effects of extreme values, we performed 1% and 99% Winsorize shrinking on the continuous variables. Data selection and sorting were performed using Excel 2010, and regression analysis was performed by Stata 15.1.

### Variables measurement

We mainly explore the impact of the number of board meetings and the proportion of the number of on-site meetings on the over-investment of listed companies in China. According to the needs of this research, the dependent variable uses the related variables of over-investment, and the independent variables use the number of board meetings, the number of board communication meetings, the number of board on-site meetings and the proportion of the number of board on-site meetings.

#### Dependent variable

In order to ensure the robustness of the research results, we adopted two methods to measure the over-investment. The first method is to refer to the research of Richardson [32], we use the model (*) to estimate the normal investment level, and then use the residuals obtained by the model regression to measure the over-investment. The absolute value of indicates the over-investment with a residual greater than 0.


$$Invest_{i,t=}a_{0}+a_{1}Size_{i,t‐1}+a_{2}Lev_{i,t‐1}+a_{3}Cash_{i,t‐1}+a_{4}Tq_{i,t‐1}+a_{5}Listage_{i,t‐1}+a_{6}Return_{i,t‐1}+a_{7}Invest_{i,t‐1}+\sum Ind+\sum Year+\varepsilon (*)$$

Invest: The cash paid for the purchase and construction of fixed assets, intangible assets and other long-term assets minus the cash recovered from the disposal of fixed assets, intangible assets and other long-term assets is higher than the total assets at the beginning of the previous year.

Size: The size of the company, measured by the logarithm of the company’s total assets.

Lev: The asset-liability ratio.

Cash: Cash holdings, calculated using the sum of total cash at the end of the year and cash equivalents divided by total assets.

Tq: Tobin’s Q value.

Listage: The age of company list. Calculated by subtracting the company’s listing year from the inspection year.

Return: Annual return on stocks.

Ind and Year: Industry and annual dummy variables.

The second method is to take the opposite of the net cash flow of investment activities in the cash flow statement and divide it by the total assets of the current year to measure the company’s total investment. The measured value exceeds the annual industry average indicates over-investment.

#### Independent variables

The number of board meetings in various forms. There are two kinds of meeting held by the Board of Directors: Regular meeting (a meeting held at a specified time, e.g. the Board of Directors of a joint stock company shall be held at least twice a year under the company law) and Ad hoc meetings (meetings that are held irregularly and are called by the chairman at any time when necessary matters are met). According to the provisions of the company law in China, each listed company must convene at least two board meetings during one year. There are no specific requirements for the form of board meetings, and there are two main methods used in practice, on-site meeting, and communications meeting. The number of board meetings intuitively reflects the strength of the board’s behavior [4]. Board meetings are a specific and critical issue that cannot be ignored in corporate governance [5]. Decisions on major company matters are all achieved through board meetings. The number of board meetings in various forms has a direct impact on the company’s investment activities.

Proportion of on-site meetings: It is defined as the proportion of the number of board meetings held by the company in the form of on-site meetings in a year to the total number of board meetings in the year. Different meeting forms directly determine the quality of decision-making, which affects the survival and development of the company. We believe that different meeting forms will have different effects on the company’s investment behavior.

#### Control variables

In order to ensure the accuracy and validity of the hypothesis test results, referring to previous research, the following control variables were selected: Year, Indus, Age, Size, Leve, Growth, Eps, difout, state, Frshr, Bsize, Outra, Comp, dual, Commeete, Dshr (Table 1).

**Table 1:** Definitions of variables.

Variables	Symbols	Definitions
Board meetings	***Mt***	Total number of board meetings during the year
Number of communication meetings	***Temt***	Number of communication meetings during the year
Number of on-site meetings	***Osmt***	Number of on-site meetings during the year
Proportion of on-site meetings	***Osmtra***	Number of on-site meetings during the year / Total number of board meetings during the year
Over-investment	***Overinv1***	If the residuals are greater than 0, it is assigned according to the actual value; if the residuals are less than or equal to 0
Independent directors in different places	***Difout***	The independent director’s residence is the same as the company’s location: 0, the difference: 1
Company achievements	***Eps***	Earnings per share = net profit / total shares
Board size	***Bsize***	The number of directors
Proportion of independent directors	***Outra***	Independent directors as a percentage of the total directors
Independent Director Allowance	***Comp***	Natural log of independent director’s allowance
The situation of two positions	***Dual***	Whether the chairman and the general manager are the same person, 0 = the same person; 1 = different one
The number of the committees of the Board of Directors	***Commeete***	The number of the committees of the Board of Directors
Board shareholding ratio	***Dshr***	The sum of the shareholding ratio of board members
Equity concentration	***Frshr***	The proportion of shares held by the largest shareholder in the total
Company Size	***Size***	Natural log of the company’s total assets
The asset-liability ratio	***Leve***	Total liabilities / total assets
The growth rate of main business	***Growth***	(The main business income this year-The main business income of last year) / The main business income at the beginning of this year
Company age	***Age***	Company listing years
Nature of equity	***State***	State-owned:1, private: 0
Industry	***Ind***	The industry
Year	***Year***	The years

### The model

In order to test the impact of the number of board meetings, the number of on-site meetings, the number of communication meetings on the over-investment (Hypothesis 1). The following models are used:

*Overinv_i =_ a_0_+a_1_Mt_i_+a_2_Difout_i_+a_3_Eps_i_+a_4_Bsize_i_+a_5_Outra_i_+a_6_Comp_i_+a_7_Dual_i_+a_8_Commeete_i_+a_9_Dshr_i_+a_10_Frshr_i_+a_11_Size_i_+a_12_Leve_i_+a_13_Growth_i_+a_14_Age_i_+a_15_State_i_ +∑Ind+∑Year+ε*

*Overinv_i =_ a_0_+a_1_Temt_i_+a_2_Difout_i_+a_3_Eps_i_+a_4_Bsize_i_+a_5_Outra_i_+a_6_Comp_i_+a_7_Dual_i_+a_8_Commeete_i_+a_9_Dshr_i_+a_10_Frshr_i_+a_11_Size_i_+a_12_Leve_i_+a_13_Growth_i_+a_14_Age_i_+a_15_State_i_ +∑Ind+∑Year+ε*

*Overinv_i =_ a_0_+a_1_Osmt_i_+a_2_Difout_i_+a_3_Eps_i_+a_4_Bsize_i_+a_5_Outra_i_+a_6_Comp_i_+a_7_Dual_i_+a_8_Commeete_i_+a_9_Dshr_i_+a_10_Frshr_i_+a_11_Size_i_+a_12_Leve_i_+a_13_Growth_i_+a_14_Age_i_+a_15_State_i_ +∑Ind+∑Year+ε*

In order to test the impact of the proportion of the number of on-site meetings on the over-investment (Hypothesis 2). The following model is used:

*Overinv_i =_ a_0_+a_1_Osmtra_i_+a_2_Difout_i_+a_3_Eps_i_+a_4_Bsize_i_+a_5_Outra_i_+a_6_Comp_i_+a_7_Dual_i_+a_8_Commeete_i_+a_9_Dshr_i_+a_10_Frshr_i_+a_11_Size_i_+a_12_Leve_i_+a_13_Growth_i_+a_14_Age_i_+a_15_State_i_ +∑Ind+∑Year+ε*

## Empirical results

### Descriptive statistics

Table 2 is a descriptive statistical result of the number of board meetings, the format of board meetings, and over-investment of the samples. It can be seen from Table 2 that the mean annual number of board meetings is 9.602, which is higher than the mean number of 7.45 meetings in the US capital market [7].

**Table 2:** Descriptive statistics.

Variable	Obs	Median	Mean	Std.Dev.	Min	Max	Skewness	Kurtosis
Overinv1	26155	0	0.019	0.048	0	0.942	5.566	54.681
Mt	26155	9	9.602	4.11	2	77	2.216	15.970
Temt	26155	4	4.73	4.581	0	55	1.740	10.206
Osmt	26155	4	4.871	3.853	0	68	1.596	9.3648
Osmtra	26155	0.5	0.529	0.342	0	1	0.164	1.598
Difout	26155	1	0.51	0.5	0	1	-0.041	1.002
Eps	26155	0.295	0.392	0.661	-7.486	30.114	8.599	283.429
Bsize	26155	9	8.756	1.773	5	19	0.823	5.7136
Outra	26155	0.333	0.373	0.054	0.333	0.75	1.649	6.372
Comp	26155	12.100	12.017	1.426	0	16.118	-7.141	60.623
Dual	26155	1	0.738	0.44	0	1	-1.080	2.1682
Commeete	26155	4	3.859	0.482	0	6	-3.684	19.855
Dshr	26155	0.04	11.897	19.328	0	89.18	1.474	3.8681
Frshr	26155	33.75	35.555	15.176	2.2	89.99	0.485	2.7654
Size	26155	21.814	21.995	1.31	14.942	28.509	0.814	4.203
Leve	26155	0.4239	0.429	0.21	0.007	0.997	0.136	2.188
Growth	26155	0.111	0.236	0.515	-0.896	9.262	6.173	65.929
Age	26155	8	9.126	6.793	0	28	0.393	2.0272
State	26155	0	0.399	0.49	0	1	0.4131	1.1706

### Regression analysis

In order to test the relationship between the number of board meetings and over-investment, we conduct regression tests on the number of board meetings, the number of communication meetings, the number of on-site meetings, the proportion of on-site meetings and over-investment. Test their relationship based on different meeting forms.

Hypothesis 1a, 1b, 1c, hypothesis 2 test.

Table 3 reports the OLS regression results of the number of board meetings, the number of communication meetings, the number of on-site meetings and the over-investment. The results are overall significant. The number of board meetings and the over-investment are significantly positively correlated at the 1% level, indicating that when the number of board meetings increases, over-investment increases. The number of communication meetings and the over-investment are significantly positively correlated at the 1% level, indicating that when the number of communication meetings increases, over-investment increases. The number of on-site meetings and the over-investment are significantly positively correlated at 1% level, indicating that when the number of on-site meetings increases, over-investment increases. The increase in the number of board meetings may indicate that the company is facing a more complex operating environment, the number of matters that require the board to make decisions may increase, and more decisions may be made about investment. It may also be due to the decline in business performance of the company, and the board may choose to increase investing to improve the company’s performance. It may also because the managers for their own benefit, the over-investment may become their "buy and build empire" arrangement, which needs to be discussed and approved by more frequent board meetings. In summary, the hypotheses 1a, 1b, 1c of this study have been verified.

**Table 3:** The regression results of the number of board meetings, the number of communication meetings, the number and proportion of on-site meetings, and the over-investment.

Explanatory variable	(1)	(2)	(3)	(4)
	Overinv1	Overinv1	Overinv1	Overinv1
Mt	0.0625***			
	(8.03)			
Temt		0.0283***		
		(3.94)		
Osmt			0.0334***	
			(4.01)	
Osmtra				-0.278***
				(-2.85)
Eps	0.209***	0.201***	0.194***	0.198***
	(4.31)	(4.14)	(4.00)	(4.07)
Difout	0.0209	-0.00538	0.0312	-0.00835
	(0.35)	(-0.09)	(0.52)	(-0.14)
Bsize	0.00868	0.000572	0.00690	-0.000925
	(0.42)	(0.03)	(0.33)	(-0.04)
Outra	0.330	0.320	0.375	0.335
	(0.53)	(0.51)	(0.60)	(0.53)
Comp	0.0143	0.0155	0.0193	0.0162
	(0.67)	(0.73)	(0.91)	(0.77)
Dual	-0.197***	-0.194***	-0.196***	-0.194***
	(-2.78)	(-2.74)	(-2.77)	(-2.73)
Commeete	0.103*	0.110*	0.109*	0.112*
	(1.66)	(1.77)	(1.74)	(1.79)
Dshr	-0.00318	-0.00257	-0.00322	-0.00243
	(-1.57)	(-1.27)	(-1.59)	(-1.20)
Frshr	-0.00470**	-0.00565***	-0.00575***	-0.00598***
	(-2.18)	(-2.63)	(-2.68)	(-2.78)
Size	-0.0279	0.00728	0.0156	0.0205
	(-0.85)	(0.22)	(0.48)	(0.63)
Leve	1.316***	1.442***	1.460***	1.483***
	(7.09)	(7.79)	(7.90)	(8.03)
Growth	1.378***	1.408***	1.393***	1.418***
	(22.55)	(23.06)	(22.76)	(23.19)
Age	0.0147**	0.0131**	0.0165***	0.0132**
	(2.46)	(2.18)	(2.76)	(2.19)
State	-0.290***	-0.323***	-0.330***	-0.336***
	(-3.79)	(-4.23)	(-4.32)	(-4.39)
Year	control	control	control	control
Ind	control	control	control	control
Constant term	1.181	1.073	0.497	1.063
	(1.44)	(1.30)	(0.61)	(1.28)
N	26155	26155	26155	26155
R2	0.046	0.044	0.044	0.044

**Note.** The t statistic of the regression coefficient is in parentheses; it controls the industry and annual dummy variables.

T statistics in parentheses

* p < 0.1

** p < 0.05

*** p < 0.01.

In order to ensure the robustness of the research conclusions, the standard errors of all tests are adjusted by heteroscedasticity and industry level clustering.

Table 3 also reports the OLS regression results of the proportion of on-site meetings and the over-investment. The results are overall significant. The proportion of on-site meetings and the over-investment were significantly negatively correlated at the 1% level, indicating that when the proportion of on-site meetings increased, the over-investment decreased. Given other conditions unchanged, if the ratio of on-site meeting increases by one standard deviation, the over investment in the current period will decrease by 0.095. Compared with the standard deviation of over investment of 0.048, this effect cannot be ignored. This result shows that the more on-site meetings are conducive to the communication between directors, thereby suppressing the company’s over-investment. This is mainly because when the board meeting takes the form of on-site meeting, more information can be shared among board members, and communication will be more convenient. These will make the directors discuss the decision more fully and make the decision more in the interests of shareholders. In addition, it is mainly because holding on-site meetings makes the Board of Directors more fully supervise the managers. It reduces the opportunistic behavior of the managers, and thus inhibit the company’s over-investment. In summary, Hypothesis 2 of this study is verified.

### Further analysis

According to whether the company has a controlling shareholder, the data is divided into two groups for statistical analysis.

The OLS regression results as follows:

Table 4 reports the OLS regression results of the number and proportion of on-site meetings and the over-investment when the company has a controlling shareholder or has not a controlling shareholder.

**Table 4:** Whether the company has a controlling shareholder.

Explanatory variable	(YES)	(NO)	(TES)	(NO)
	Overinv1	Overinv1	Overinv1	Overinv1
Osmt	0.0395**	0.0330***		
	(1.96)	(3.61)		
Osmtra			-0.227	-0.270**
			(-1.01)	(-2.50)
Eps	0.146**	0.229***	0.149**	0.234***
	(1.97)	(3.70)	(2.01)	(3.77)
Difout	-0.267**	0.0949	-0.291**	0.0539
	(-1.99)	(1.41)	(-2.17)	(0.80)
Bsize	0.0290	0.00782	0.0185	0.000342
	(0.69)	(0.33)	(0.44)	(0.01)
Outra	0.636	0.784	0.630	0.719
	(0.50)	(1.09)	(0.49)	(1.00)
Comp	0.00564	0.0253	0.00473	0.0218
	(0.13)	(1.06)	(0.11)	(0.91)
Dual	-0.382**	-0.149*	-0.387**	-0.145*
	(-2.07)	(-1.92)	(-2.10)	(-1.87)
Commeete	-0.246**	0.184**	-0.236*	0.186**
	(-2.03)	(2.55)	(-1.95)	(2.58)
Dshr	-0.00792*	-0.00225	-0.00748*	-0.00138
	(-1.86)	(-0.97)	(-1.76)	(-0.59)
Size	-0.135**	0.0554	-0.133**	0.0598
	(-2.03)	(1.47)	(-2.00)	(1.59)
Leve	2.554***	1.338***	2.537***	1.373***
	(5.81)	(6.50)	(5.76)	(6.68)
Growth	1.244***	1.394***	1.277***	1.418***
	(7.79)	(20.92)	(7.99)	(21.30)
Age	0.00909	0.0180***	0.00641	0.0149**
	(0.72)	(2.65)	(0.51)	(2.19)
State	-0.288*	-0.340***	-0.274	-0.352***
	(-1.66)	(-4.02)	(-1.58)	(-4.16)
Year	control	control	control	control
Ind	control	control	control	control
Constant term	2.975*	-0.512	3.500**	0.0779
	(1.86)	(-0.53)	(2.16)	(0.08)
N	4389	21766	4389	21766
R2	0.093	0.045	0.092	0.044

**Note.** The t statistic of the regression coefficient is in parentheses; it controls the industry and annual dummy variables.

T statistics in parentheses

* p < 0.1

** p < 0.05

*** p < 0.01.

In order to ensure the robustness of the research conclusions, the standard errors of all tests are adjusted by heteroscedasticity and industry level clustering.

Regarding the regression of the number of on-site meetings of the Board of Directors and the over-investment, the results show that the number of on-site meetings and over-investment are positively correlated regardless of the company has a controlling shareholder or has not a controlling shareholder. It is consistent with the results of assumption 1.

Regarding the regression of the proportion of on-site meetings and the over-investment, the results show that the regression results of the proportion of on-site meetings and the over-investment are not significant overall when the company has a controlling shareholder, the increase in the proportion of on-site meetings will weaken the over-investment. It is mainly because the controlling shareholder can easily use his position to control the Board of Directors and the Board of Supervisors, thereby achieving superior control in corporate governance. It is difficult to form an effective internal control and check-and-balance mechanism in the case of highly concentrated equity. When the company does not have a controlling shareholder, the OLS regression results are overall significant. The proportion of on-site meetings and the over-investment are significantly negatively correlated at a 5% level. The increase in the proportion of on-site meetings can inhibit the over-investment. This is mainly because when the company does not have a controlling shareholder, the Board of Directors will not appear to be a dominant phenomenon, and the directors can communicate more fully at the board meeting. Thereby the on-site meetings can inhibit the company’s over-investment.

According to the situation that one manager holds both the position of general manager and the position of president of Board of Directors (duality of CEO), the data is divided into two groups for statistical analysis.

The OLS regression results are as follows:

Table 5 reports the OLS regression results of the number and the proportion of on-site meetings and the over-investment when the company is duality or no.

**Table 5:** The company is duality or no.

Explanatory variable	(NO)	(YES)	(NO)	(YES)
	Overinv1	Overinv1	Overinv1	Overinv1
Osmt	0.0216**	0.0551***		
	(2.28)	(3.23)		
Osmtra			-0.310***	-0.198
			(-2.75)	(-1.02)
Eps	0.201***	0.110	0.207***	0.109
	(3.50)	(1.20)	(3.60)	(1.19)
Difout	-0.0265	0.150	-0.0557	0.0819
	(-0.39)	(1.18)	(-0.81)	(0.65)
Bsize	0.0210	-0.0186	0.0152	-0.0335
	(0.93)	(-0.36)	(0.67)	(-0.65)
Outra	0.0456	0.988	0.0372	0.801
	(0.06)	(0.74)	(0.05)	(0.60)
Comp	0.0336	-0.0550	0.0308	-0.0583
	(1.46)	(-1.07)	(1.34)	(-1.14)
Commeete	0.122*	0.134	0.125*	0.137
	(1.76)	(0.98)	(1.79)	(1.00)
Dshr	-0.00251	-0.00421	-0.00170	-0.00362
	(-0.97)	(-1.24)	(-0.65)	(-1.07)
Frshr	-0.00922***	0.00697	-0.00941***	0.00683
	(-3.79)	(1.50)	(-3.87)	(1.47)
Size	0.00920	-0.00530	0.0118	0.00328
	(0.25)	(-0.07)	(0.33)	(0.04)
Leve	1.139***	2.543***	1.140***	2.647***
	(5.43)	(6.48)	(5.43)	(6.76)
Growth	1.898***	0.700***	1.919***	0.728***
	(24.68)	(6.72)	(24.99)	(6.97)
Age	0.0211***	0.00813	0.0185***	0.00294
	(3.17)	(0.58)	(2.77)	(0.21)
State	-0.213**	-0.824***	-0.212**	-0.848***
	(-2.55)	(-4.40)	(-2.53)	(-4.53)
Year	control	control	control	control
Ind	control	control	control	control
Constant term	0.448	1.342	0.960	2.039
	(0.50)	(0.64)	(1.06)	(0.96)
N	19295	6860	19295	6860
R2	0.059	0.052	0.059	0.051

**Note.** The t statistic of the regression coefficient is in parentheses; it controls the industry and annual dummy variables.

T statistics in parentheses

* p < 0.1

** p < 0.05

*** p < 0.01.

In order to ensure the robustness of the research conclusions, the standard errors of all tests are adjusted by heteroscedasticity and industry level clustering.

Regarding the regression of the number of on-site meetings and the over-investment, the results show that the number of on-site meetings and the over-investment are positively correlated regardless of duality or no. It is consistent with the results of Hypothesis 1. However, when the two positions are separated is weaker than when the two positions are held concurrently. The results indicate that when the company in the state which the two positions are separated, the effect of on-site meetings is stronger than when the two positions are held concurrently.

Regarding the regression of the proportion on-site meetings and over-investment, the results show that when the company is duality, the regression results are not significant overall. It shows that when the proportion of on-site meetings increases when the company is not duality, the inhibitory effect on excessive investment will be weakened. This may be due to the increase in the degree of entrusted agency when the company is duality, the general manager supervising himself, and excessive concentration of power. When holding a board meeting, the general manager is likely to use his power to influence the voting results of the board meeting, and the profit-seeking nature of managers will make them tend to overinvest. When the company is not duality, the proportion on-site meetings of the Board of Directors and over-investment OLS regression results are overall significant and significantly negatively correlated at the 5% level. When the company implements the separation of the two positions, the proportion of on-site meetings have an inhibitory effect on over-investment. This may be due to the fact that when the company is not duality, the influence of the manager on the Board of Directors is relatively small, and the independence of the Board of Directors increases. The on-site meeting can communicate more fully, and the directors can better supervise the managers to inhibit the managers from investing in projects with negative net cash flow to maximize their own interests.

According to different proportion of independent directors where the ratio of independent directors of the board is below 0.5 and above 0.5. The data is divided into two groups for statistical analysis.

The OLS regression results are as follows:

Table 6 reports the OLS regression results of the number and the proportion of on-site meetings and the over-investment when the company’s independent director ratio is below 0.5 or above 0.5.

**Table 6:** Different proportion of independent directors.

Explanatory variable	(Above 0.5)	(Below 0.5)	(Above 0.5)	(Below 0.5)
	Overinv1	Overinv1	Overinv1	Overinv1
Osmt	-0.0257	0.0366***		
	(-0.73)	(4.27)		
Osmtra			-0.879**	-0.251**
			(-2.05)	(-2.50)
Eps	0.0715	0.228***	0.0786	0.233***
	(0.66)	(4.21)	(0.72)	(4.29)
Difout	-0.173	0.0438	-0.222	0.00447
	(-0.65)	(0.70)	(-0.83)	(0.07)
Bsize	0.0371	-0.00260	0.0312	-0.0103
	(0.38)	(-0.13)	(0.32)	(-0.52)
Comp	-0.189*	0.0275	-0.193*	0.0245
	(-1.72)	(1.27)	(-1.76)	(1.13)
Dual	-0.242	-0.206***	-0.221	-0.205***
	(-0.81)	(-2.81)	(-0.75)	(-2.81)
Commeete	0.119	0.112*	0.107	0.116*
	(0.38)	(1.75)	(0.34)	(1.82)
Dshr	0.00978	-0.00408*	0.00951	-0.00323
	(1.12)	(-1.96)	(1.09)	(-1.55)
Frshr	0.0101	-0.00671***	0.0108	-0.00700***
	(1.13)	(-3.02)	(1.21)	(-3.16)
Size	-0.0815	0.0275	-0.0925	0.0324
	(-0.64)	(0.81)	(-0.73)	(0.96)
Leve	2.258***	1.453***	2.233***	1.480***
	(2.60)	(7.65)	(2.59)	(7.80)
Growth	1.712***	1.381***	1.729***	1.406***
	(5.98)	(22.00)	(6.07)	(22.40)
Age	-0.0174	0.0166***	-0.0237	0.0134**
	(-0.63)	(2.69)	(-0.86)	(2.17)
State	-0.333	-0.316***	-0.310	-0.324***
	(-0.90)	(-4.03)	(-0.84)	(-4.14)
Year	control	control	control	control
Ind	control	control	control	control
Constant term	3.657	0.414	4.567	0.961
	(1.10)	(0.50)	(1.36)	(1.15)
N	1236	24919	1236	24919
R2	0.138	0.044	0.141	0.044

**Note.** The t statistic of the regression coefficient is in parentheses; it controls the industry and annual dummy variables.

T statistics in parentheses

* p < 0.1

** p < 0.05

*** p < 0.01.

In order to ensure the robustness of the research conclusions, the standard errors of all tests are adjusted by heteroscedasticity and industry level clustering.

Regarding the regression of the number of on-site meetings and the over-investment, the results show that when the proportion of independent directors is below 0.5, the number of on-site meetings and the over-investment are significantly positively correlated at the 1% level. It is negatively correlated, but not significant when the proportion of independent directors is above 0.5. This shows that independent directors can effectively curb the over-investment behavior of the company.

Regarding the regression of the proportion board on-site meetings and the over-investment, the results show that the number of on-site meetings and the over-investment are significantly negatively correlated at the 5% level when the proportion of independent directors is below 0.5 or When the proportion of independent directors is 0.5 or above. This shows that due to the externality of independent directors, the managers can be more effectively supervised. When on-site meetings are used, independent directors have an inhibitory effect on excessive investment, and the higher the proportion of independent directors, the more significant the inhibitory effect.

According to The different nature of company’s equity, the data is divided into two groups: the state-owned enterprises and the private enterprises.

The OLS regression results are as follows:

Table 7 reports the OLS regression results of the number and proportion of on-site meetings and the over-investment when the company is state-owned and privately held.

**Table 7:** The different nature of company’s equity.

Explanatory variable	(Private)	(State-owned)	(Private)	(State-owned)
	Overinv1	Overinv1	Overinv1	Overinv1
Osmt	0.0411***	0.0148		
	(3.87)	(1.11)		
Osmtra			-0.207*	-0.299*
			(-1.65)	(-1.91)
Eps	0.225***	0.169***	0.226***	0.175***
	(2.88)	(2.84)	(2.88)	(2.93)
Difout	0.0318	0.0402	-0.0107	0.0179
	(0.39)	(0.45)	(-0.13)	(0.20)
Bsize	-0.0315	0.0552**	-0.0436	0.0522**
	(-0.97)	(2.09)	(-1.35)	(1.98)
Outra	1.118	-0.705	0.978	-0.684
	(1.24)	(-0.80)	(1.08)	(-0.77)
Comp	0.0214	0.0194	0.0149	0.0187
	(0.66)	(0.73)	(0.46)	(0.70)
Dual	-0.265***	0.00723	-0.262***	0.0118
	(-3.10)	(0.05)	(-3.06)	(0.09)
Commeete	0.196**	-0.0196	0.194**	-0.0145
	(2.16)	(-0.23)	(2.13)	(-0.17)
Dshr	-0.000783	-0.0212**	-0.000263	-0.0209**
	(-0.34)	(-2.53)	(-0.11)	(-2.50)
Frshr	9.08e-09	-0.0110***	-0.000445	-0.0109***
	(0.00)	(-3.53)	(-0.15)	(-3.51)
Size	0.166***	-0.162***	0.175***	-0.163***
	(3.40)	(-3.73)	(3.58)	(-3.75)
Leve	1.480***	1.496***	1.523***	1.507***
	(5.90)	(5.42)	(6.08)	(5.46)
Growth	1.059***	3.251***	1.083***	3.266***
	(15.07)	(22.72)	(15.40)	(22.88)
Age	0.0170*	0.0301***	0.0118	0.0288***
	(1.93)	(3.59)	(1.34)	(3.44)
Year	control	control	control	control
Ind	control	control	control	control
Constant term	-2.594**	3.945***	-2.000	4.375***
	(-2.00)	(3.74)	(-1.53)	(4.10)
N	15723	10432	15723	10432
R2	0.045	0.089	0.045	0.089

**Note.** The t statistic of the regression coefficient is in parentheses; it controls the industry and annual dummy variables.

T statistics in parentheses

* p < 0.1

** p < 0.05

*** p < 0.01.

In order to ensure the robustness of the research conclusions, the standard errors of all tests are adjusted by heteroscedasticity and industry level clustering.

Regarding the regression of the number of on-site meetings and the over-investment, the results show that the number of on-site meetings and the over-investment are significantly positively correlated at the 1% level when the company is privately held. The number of on-site meetings is positively correlated with and the over-investment when the company is state-owned, but not significant. Compared with private companies, state-owned companies are more cautious in investment behavior, even if there are more board meetings, there will be no more over-investment.

Regarding the regression of the proportion on-site meetings and the over-investment, the results show that the proportion on-site meetings and the over-investment are not significantly correlated when the company is private. The proportion on-site meetings and the over-investment are significantly negatively correlated at 10% when the company is state-owned. It shows that when the company is a state-owned holding company, the effect of on-site meetings is better than when the company is a private holding company. On site meeting is more effective in state-owned company’ decision-making. This is due to the more effective supervision mechanism of state-owned companies. At the same time, it also reflects that there are more factors influencing the investment behavior of private companies.

### Robustness test

In order to ensure the robustness of the empirical results, we conducted a robustness test in two ways.

Change the variable calculation method. This paper uses Richardson model to calculate the measurement method used by enterprises when they overinvest. Tobin Q is replaced by the growth rate of sales revenue, and then overinv2 is obtained to replace overinv2 for robustness test. The overall results showed no difference, which verified the hypothesis of the research again.

Table 8 reports the Logit regression results of the number of board meetings, the number of communication meetings, the number of on-site meetings, the proportion on-site meetings and the over-investment. The hypotheses 1a, 1b, 1c, 2 of this study have been verified.

**Table 8:** The regression results of robustness test1.

Explanatory variable	(1)	(2)	(3)	(4)
	Overinv2	Overinv2	Overinv2	Overinv2
Mt	0.0528***			
	(7.06)			
Temt		0.0231***		
		(3.34)		
Osmt			0.0293***	
			(3.67)	
Osmtra				-0.251***
				(-2.68)
Eps	0.198***	0.190***	0.185***	0.188***
	(4.24)	(4.09)	(3.97)	(4.04)
Difout	0.0321	0.0103	0.0416	0.00643
	(0.56)	(0.18)	(0.72)	(0.11)
Bsize	0.00711	0.000294	0.00577	-0.00116
	(0.36)	(0.01)	(0.29)	(-0.06)
Outra	0.381	0.372	0.419	0.384
	(0.63)	(0.62)	(0.70)	(0.64)
Comp	0.0131	0.0142	0.0174	0.0147
	(0.64)	(0.70)	(0.85)	(0.72)
Dual	-0.173**	-0.171**	-0.173**	-0.171**
	(-2.55)	(-2.51)	(-2.53)	(-2.50)
Commeete	0.107*	0.113*	0.112*	0.115*
	(1.79)	(1.89)	(1.87)	(1.91)
Dshr	-0.00321*	-0.00270	-0.00326*	-0.00256
	(-1.65)	(-1.39)	(-1.67)	(-1.31)
Frshr	-0.00434**	-0.00515**	-0.00522**	-0.00542***
	(-2.10)	(-2.49)	(-2.53)	(-2.63)
Size	0.0149	0.0451	0.0514*	0.0556*
	(0.47)	(1.44)	(1.65)	(1.79)
Leve	1.219***	1.327***	1.339***	1.360***
	(6.83)	(7.46)	(7.54)	(7.66)
Growth	1.117***	1.142***	1.128***	1.151***
	(19.01)	(19.46)	(19.18)	(19.58)
Age	0.00324	0.00191	0.00486	0.00185
	(0.56)	(0.33)	(0.84)	(0.32)
State	-0.251***	-0.279***	-0.284***	-0.289***
	(-3.41)	(-3.80)	(-3.87)	(-3.94)
Year	control	control	control	control
Ind	control	control	control	control
Constant term	0.365	0.263	-0.219	0.287
	(0.46)	(0.33)	(-0.28)	(0.36)
N	26155	26155	26155	26155
R2	0.038	0.036	0.036	0.036

**Note.** The z statistic of the regression coefficient is in parentheses; it controls the industry and annual dummy variables.

Z statistics in parentheses

* p < 0.1

** p < 0.05

*** p < 0.01.

In order to ensure the robustness of the research conclusions, the standard errors of all tests are adjusted by heteroscedasticity and industry level clustering.

The samples was changed for robustness test, and the samples from 2008 to 2017 were selected for empirical test. Table 9 reports the results showed no difference as a whole, which verified the hypothesis of this research again.

**Table 9:** The regression results of robustness test2.

Explanatory variable	(1)	(2)	(3)	(4)
	Overinv1	Overinv1	Overinv1	Overinv1
Mt	0.0674***			
	(8.07)			
Temt		0.0327***		
		(4.22)		
Osmt			0.0343***	
			(3.80)	
Osmtra				-0.297***
				(-2.83)
Eps	0.169***	0.159***	0.150***	0.155***
	(2.99)	(2.81)	(2.65)	(2.73)
Difout	0.0952	0.0654	0.105	0.0634
	(1.46)	(1.00)	(1.61)	(0.97)
Bsize	0.0155	0.00689	0.0136	0.00550
	(0.69)	(0.31)	(0.60)	(0.24)
Outra	0.650	0.612	0.686	0.624
	(0.96)	(0.91)	(1.02)	(0.92)
Comp	0.00975	0.0102	0.0154	0.0114
	(0.43)	(0.45)	(0.67)	(0.50)
Dual	-0.225***	-0.224***	-0.222***	-0.223***
	(-2.95)	(-2.92)	(-2.90)	(-2.91)
Commeete	0.0270	0.0328	0.0337	0.0352
	(0.36)	(0.44)	(0.45)	(0.47)
Dshr	-0.00387*	-0.00311	-0.00385*	-0.00297
	(-1.78)	(-1.43)	(-1.77)	(-1.37)
Frshr	-0.00490**	-0.00584**	-0.00606***	-0.00623***
	(-2.12)	(-2.53)	(-2.63)	(-2.70)
Size	-0.0255	0.0102	0.0213	0.0251
	(-0.71)	(0.29)	(0.61)	(0.71)
Leve	1.248***	1.380***	1.399***	1.427***
	(6.23)	(6.91)	(7.01)	(7.16)
Growth	1.152***	1.185***	1.167***	1.196***
	(18.27)	(18.82)	(18.48)	(18.95)
Age	0.0185***	0.0168**	0.0207***	0.0171***
	(2.83)	(2.56)	(3.16)	(2.60)
State	-0.275***	-0.310***	-0.318***	-0.324***
	(-3.34)	(-3.77)	(-3.87)	(-3.94)
Year	control	control	control	control
Ind	control	control	control	control
Constant term	1.285	1.232	0.574	1.201
	(1.43)	(1.36)	(0.64)	(1.32)
*N*	21880	21880	21880	21880
*R*^2^	0.042	0.040	0.040	0.040

**Note.** The t statistic of the regression coefficient is in parentheses; it controls the industry and annual dummy variables.

T statistics in parentheses

* p < 0.1

** p < 0.05

*** p < 0.01.

In order to ensure the robustness of the research conclusions, the standard errors of all tests are adjusted by heteroscedasticity and industry level clustering.

## Conclusions

This paper studies the relationship between the number of board meetings, the proportion of on-site meetings and the over-investment. Based on the data of listed companies from 2007 to 2017, take the number of board meetings, on-site meetings, communication meetings, and the proportion on-site meetings of the Board of Directors as independent variable, and the company’s over-investment is the dependent variable. The analysis found that the decision of the on-site board meeting of the Board of Directors will inhibit the over-investment to a certain extent. Further research found that when the company has a controlling shareholder and the duality, the on-site meetings’ inhibitory effect on over-investment will be weakened. The presence of independent directors has a certain effect on suppressing over-investment. The higher the percentage of independent directors, the more obvious inhibitory effect is. The comparison between the state-owned companies and the private companies found that the on-site meeting’s inhibitory effect on over-investment is more obvious in state-owned enterprises.

Board meetings are an important way for the Board of Directors to make decisions and exercise supervisory power over company matters. The quality of board meetings directly affects the quality of decision-making and the effectiveness of supervision. The conclusion of this article shows that the on-site meeting of the Board of Directors is conducive to making more favorable decisions and better supervision for the company. Based on the conclusions of this article, the following suggestions are made:

First, regarding the choice of the form of board meetings, for listed companies, it is necessary to recognize the importance of board meetings, establish and improve a more complete board meeting operating system, make different regulations for different matters. For decisions on major matters of the company, especially proposals requiring full communication between directors, we recommend that a board meeting be held in the form of an on-site meeting; For some procedural votes, in order to save costs, the form of communication meeting can be used appropriately, but the information required for the meeting must be sent to the directors, especially the external directors, in advance, so that they can fully understand the matters to be voted and allow the board meeting can play its due role. For the relevant regulatory authorities, they can set up a corresponding system to set a minimum requirement for the proportion of board meetings of listed companies. Regular board meetings should be stipulated as on-site meetings. Temporary meetings can be freely chosen according to the matters discussed form.

Secondly, in the internal governance structure of the company, we should strengthen the focus on independent directors, increase the status of independent directors, appropriately increase the proportion of independent directors in the Board of Directors, and provide independent directors or external directors with company-related business information in a timely manner. Reduce the occurrence of information asymmetry, so that independent directors can really play a role in the decision-making of board meetings; combined with the current split share structure reform, change the ownership structure and the dominant ownership structure, improve related laws and regulations and the articles of association of listed companies, strengthen the supervision of large shareholders, reduce the control of large shareholders on the Board of Directors, prevent them from infringing the interests of small and medium shareholders for their own interests; Increase the independence of the Board of Directors, strengthen the supervision of the Board of Directors on management, In particular, the general manager should be strictly supervised by the Board of Directors, rather than self-monitoring; Private enterprises may have fewer supervision procedures when making investment decisions than state-owned enterprises, and it may be easier to make decisions to increase investment. Therefore, the Board of Directors of private enterprises should be more cautious when making decisions and should pay more attention to on-site meeting.

Thirdly, during the COVID-19 period, most of the world is using online meetings. This, of course, include board meetings. Online meetings seem to be becoming a trend. In such a situation, on the one hand, the enterprise should also do a good job in the communication meeting, increase the information communication before, during and after the board meeting, and realize the optimal realization of the meeting information communication. On the other hand, the enterprise should also provide more information communication channels and face-to-face on-site communication opportunities for the directors of the board, and should try their best to use on-site meeting for major decision-making matters.

In short, the choice of the format of the board meeting should be determined according to the importance of the company’ss decision-making matters, and should not be affected by the structure of the Board of Directors, the control of the major shareholders, the management, the nature of equity, etc., so that the board meeting becomes a booster for the company’s development.

## Supporting information

S1 Table Correlation matrix. (DOCX)

S2 Table Nonlinear regression results of the number of board meetings and overinvestment. (DOCX)
