# Supplementary material for: Does the board’s on-site decision inhibit over-investment
Source: PLoS One. 2021 Aug 5;16(8):e0255453. doi: 10.1371/journal.pone.0255453 (PMC8341494; doi:10.1371/journal.pone.0255453)
Supplement: S1 Table — (DOCX) [file pone.0255453.s001.docx]

**S1 Correlation matrix**

| Variables | (1) | (2) | (3) | (4) | (5) | (6) | (7) | (8) | (9) | (10) | (11) | (12) | (13) | (14) | (15) | (16) | (17) | (18) |
| --- | --- | --- | --- | --- | --- | --- | --- | --- | --- | --- | --- | --- | --- | --- | --- | --- | --- | --- |
| (1) Overinv1 | 1 |  |  |  |  |  |  |  |  |  |  |  |  |  |  |  |  |  |
| (2) Mt | 0.054^***^ | 1 |  |  |  |  |  |  |  |  |  |  |  |  |  |  |  |  |
| (3) Temt | 0.006 | 0.612^***^ | 1 |  |  |  |  |  |  |  |  |  |  |  |  |  |  |  |
| (4) Osmt | 0.050^***^ | 0.339^***^ | -0.536^**^ | 1 |  |  |  |  |  |  |  |  |  |  |  |  |  |  |
| (5) Osmtra | 0.012 | -0.148^***^ | -0.804^***^ | 0.798^***^ | 1 |  |  |  |  |  |  |  |  |  |  |  |  |  |
| (6) Difout | 0.011^**^ | -0.030^***^ | 0.050^***^ | -0.092^***^ | -0.086^***^ | 1 |  |  |  |  |  |  |  |  |  |  |  |  |
| (7) Eps | 0.037^***^ | -0.008 | -0.016^***^ | 0.011^**^ | 0.026^***^ | 0.009^*^ | 1 |  |  |  |  |  |  |  |  |  |  |  |
| (8) Bsize | 0.020^***^ | -0.014^***^ | 0.009 | -0.026^***^ | -0.022^***^ | 0.039^***^ | 0.048^***^ | 1 |  |  |  |  |  |  |  |  |  |  |
| (9) Outra | -0.005 | 0.047^***^ | 0.037^***^ | 0.006 | -0.015^***^ | -0.014^***^ | -0.005 | -0.442^***^ | 1 |  |  |  |  |  |  |  |  |  |
| (10)Comp | 0.020 | 0.073^***^ | 0.110^***^ | -0.053^***^ | -0.101^***^ | 0.002 | 0.041^***^ | 0.081^***^ | 0.058^***^ | 1 |  |  |  |  |  |  |  |  |
| (11) Dual | -0.016^***^ | 0.013^***^ | 0.020^***^ | -0.01 | -0.021^***^ | 0.009 | -0.029^***^ | 0.176^***^ | -0.108^***^ | -0.002^*^ | 1 |  |  |  |  |  |  |  |
| (12)Commeete | 0.002 | 0.026^***^ | 0.061^***^ | -0.045^***^ | -0.068^***^ | 0.026^***^ | -0.008 | -0.012^**^ | 0.028^***^ | 0.034^***^ | -0.017^***^ | 1 |  |  |  |  |  |  |
| (13)Dshr | -0.004 | -0.029^***^ | -0.085^***^ | 0.070^***^ | 0.098^***^ | -0.057^***^ | 0.065^***^ | -0.211^***^ | 0.080^***^ | 0.006^*^ | -0.264^***^ | 0.038^***^ | 1 |  |  |  |  |  |
| (14)Frshr | -0.017^***^ | -0.057^***^ | -0.046^***^ | -0.005^***^ | 0.027^***^ | -0.025^***^ | 0.123^***^ | 0.029^***^ | 0.047^***^ | 0.020^***^ | 0.048^***^ | -0.032^***^ | -0.093^***^ | 1 |  |  |  |  |
| (15)Size | 0.013^***^ | 0.252^***^ | 0.261^***^ | -0.041^***^ | -0.177^***^ | 0.012^***^ | 0.187^***^ | 0.269^***^ | 0.030^***^ | 0.179^***^ | 0.171^***^ | 0.007^***^ | -0.306^***^ | 0.219^***^ | 1 |  |  |  |
| (16) Leve | 0.034^***^ | 0.226^***^ | 0.169^***^ | 0.040^***^ | -0.078^***^ | 0.027^***^ | -0.118^***^ | 0.177^***^ | -0.018^***^ | 0.041^***^ | 0.163^***^ | 0.013^***^ | -0.355^***^ | 0.062^***^ | 0.485^***^ | 1 |  |  |
| (17) Growth | 0.138 | 0.050^***^ | -0.046^***^ | 0.108^***^ | 0.112^***^ | -0.001 | 0.179^**^ | -0.042^*^ | 0.003 | -0.011^*^ | -0.105^***^ | -0.005 | 0.213^***^ | -0.015^***^ | -0.055^***^ | -0.143^***^ | 1 |  |
| (18) Age | -0.008 | 0.105^***^ | 0.221^***^ | -0.150^***^ | -0.224^***^ | 0.019^***^ | -0.082^***^ | 0.098^***^ | -0.019^***^ | 0.030^***^ | 0.212^***^ | -0.014^***^ | -0.535^***^ | -0.073^***^ | 0.348^***^ | 0.380^***^ | -0.242^***^ | 1 |
| (19) State | -0.016^***^ | -0.040^***^ | -0.005 | -0.036^***^ | -0.020^***^ | -0.059^***^ | 0.020^***^ | 0.272^***^ | -0.055^***^ | 0.004 | 0.256^***^ | -0.060^***^ | -0.461^***^ | 0.232^***^ | 0.336^***^ | 0.260^***^ | -0.152^***^ | 0.356^***^ |

**Note**

T statistics in parentheses, * p < 0.1, ** p < 0.05, *** p < 0.01.
