# Supplementary material for: Does the board’s on-site decision inhibit over-investment
Source: PLoS One. 2021 Aug 5;16(8):e0255453. doi: 10.1371/journal.pone.0255453 (PMC8341494; doi:10.1371/journal.pone.0255453)
Supplement: S2 Table — (DOCX) [file pone.0255453.s002.docx]

**S2 Nonlinear regression results of the number of board meetings and overinvestment.**

| Explanatory variable | Overinv1 | Overinv1 | Overinv1 |
| --- | --- | --- | --- |
| Mt2 | -0.0295^***^ |  |  |
|  | (-5.35) |  |  |
| Mt | 0.147^***^ |  |  |
|  | (8.35) |  |  |
| Temt2 |  | -0.0211^***^ |  |
|  |  | (-3.64) |  |
| Temt |  | 0.0667^***^ |  |
|  |  | (5.23) |  |
| Osmt2 |  |  | 0.0156^*^ |
|  |  |  | (1.88) |
| Osmt |  |  | 0.0778 |
|  |  |  | (0.49) |
| Eps | 0.213^***^ | 0.204^***^ | 0.195^***^ |
|  | (4.40) | (4.21) | (4.03) |
| Difout | 0.0192 | -0.0161 | 0.0277 |
|  | (0.32) | (-0.27) | (0.46) |
|  |  |  |  |
| Bsize | 0.0111 | -0.00109 | 0.00636 |
|  | (0.53) | (-0.05) | (0.31) |
| Outra | 0.329 | 0.325 | 0.385 |
|  | (0.53) | (0.52) | (0.61) |
| Comp | 0.0138 | 0.0145 | 0.0188 |
|  | (0.65) | (0.68) | (0.89) |
| Dual | -0.198^***^ | -0.195^***^ | -0.196^***^ |
|  | (-2.80) | (-2.75) | (-2.77) |
| Commeete | 0.0999 | 0.111^*^ | 0.110^*^ |
|  | (1.60) | (1.79) | (1.76) |
| Dshr | -0.00322 | -0.00228 | -0.00316 |
|  | (-1.59) | (-1.12) | (-1.56) |
| Frshr | -0.00478^**^ | -0.00585^***^ | -0.00567^***^ |
|  | (-2.22) | (-2.72) | (-2.64) |
| Size | -0.0322 | 0.0112 | 0.0132 |
|  | (-0.98) | (0.34) | (0.41) |
| Leve | 1.273^***^ | 1.446^***^ | 1.460^***^ |
|  | (6.85) | (7.81) | (7.90) |
| Growth | 1.375^***^ | 1.416^***^ | 1.393^***^ |
|  | (22.50) | (23.18) | (22.77) |
| Age | 0.0143^**^ | 0.0121^**^ | 0.0163^***^ |
|  | (2.39) | (2.01) | (2.72) |
| State | -0.272^***^ | -0.324^***^ | -0.328^***^ |
|  | (-3.55) | (-4.23) | (-4.29) |
| Ind | YES | YES | YES |
| Year | YES | YES | YES |
| Constant term | 0.765 | 0.992 | 0.624 |
|  | (0.93) | (1.20) | (0.76) |
| *N* | 26155 | 26155 | 26155 |
| *R*^2^ | 0.147 | 0.144 | 0.144 |

**Note**

The t statistic of the regression coefficient is in parentheses; it controls the industry and annual dummy variables.

T statistics in parentheses, * p < 0.1, ** p < 0.05, *** p < 0.01.

In order to ensure the robustness of the research conclusions, the standard errors of all tests are adjusted by heteroscedasticity and industry level clustering.
